# Proteomic Profiling Reveals That Resveratrol Inhibits HSP27 Expression and Sensitizes Breast Cancer Cells to Doxorubicin Therapy

**DOI:** 10.1371/journal.pone.0064378

**Published:** 2013-05-27

**Authors:** José Díaz-Chávez, Miguel A. Fonseca-Sánchez, Elena Arechaga-Ocampo, Ali Flores-Pérez, Yadira Palacios-Rodríguez, Guadalupe Domínguez-Gómez, Laurence A. Marchat, Lizeth Fuentes-Mera, Guillermo Mendoza-Hernández, Patricio Gariglio, César López-Camarillo

**Affiliations:** 1 Carcinogenesis Laboratory, National Institute of Cancerology, Mexico City, Mexico; 2 Oncogenomics and Cancer Proteomics Laboratory, Genomics Sciences Program, Autonomous University of Mexico City, Mexico City, Mexico; 3 Virus and Cancer Laboratory, National Institute of Cancerology, Mexico City, Mexico; 4 Molecular Biomedicine Program and Biotechnology Network, National School of Medicine and Homeopathy, National Polytechnic Institute, Mexico City, Mexico; 5 Molecular Biology and Histocompatibility Laboratory, General “Dr. Manuel Gea González” Hospital, Mexico City, Mexico; 6 Medicine Faculty, Autonomous National University of Mexico, Mexico City, Mexico; 7 Genetics and Molecular Biology Department, Center of Research and Advances Studies, Mexico City, Mexico; National Institutes of Health, United States of America

## Abstract

The use of chemopreventive natural compounds represents a promising strategy in the search for novel therapeutic agents in cancer. Resveratrol (3,4′,5-trans-trihydroxystilbilene) is a dietary polyphenol found in fruits, vegetables and medicinal plants that exhibits chemopreventive and antitumor effects. In this study, we searched for modulated proteins with preventive or therapeutic potential in MCF-7 breast cancer cells exposed to resveratrol. Using two-dimensional electrophoresis we found significant changes (FC >2.0; p≤0.05) in the expression of 16 proteins in resveratrol-treated MCF-7 cells. Six down-regulated proteins were identified by tandem mass spectrometry (ESI-MS/MS) as heat shock protein 27 (HSP27), translationally-controlled tumor protein**,** peroxiredoxin-6, stress-induced-phosphoprotein-1, pyridoxine-5′-phosphate oxidase-1 and hypoxanthine-guanine phosphoribosyl transferase; whereas one up-regulated protein was identified as triosephosphate isomerase. Particularly, HSP27 overexpression has been associated to apoptosis inhibition and resistance of human cancer cells to therapy. Consistently, we demonstrated that resveratrol induces apoptosis in MCF-7 cells. Apoptosis was associated with a significant increase in mitochondrial permeability transition, cytochrome *c* release in cytoplasm, and caspases -3 and -9 independent cell death. Then, we evaluated the chemosensitization effect of increasing concentrations of resveratrol in combination with doxorubicin anti-neoplastic agent *in vitro*. We found that resveratrol effectively sensitize MCF-7 cells to cytotoxic therapy. Next, we evaluated the relevance of HSP27 targeted inhibition in therapy effectiveness. Results evidenced that HSP27 inhibition using RNA interference enhances the cytotoxicity of doxorubicin. In conclusion, our data indicate that resveratrol may improve the therapeutic effects of doxorubicin in part by cell death induction. We propose that potential modulation of HSP27 levels using natural alternative agents, as resveratrol, may be an effective adjuvant in breast cancer therapy.

## Introduction

Breast cancer is the most frequently diagnosed cancer and the leading cause of cancer death in women worldwide, accounting for 23% (1.38 million) of the total new cancer cases and 14% (485,400) of the total cancer deaths [1]. A large body of epidemiologic data supports the fact that diet and nutrition play a key role in carcinogenesis [2], [3], as reflected in the global differences in breast cancer incidence worldwide [4]. High fat, meat-based and low fiber diets are associated to high breast cancer incidence rates whereas the lowest rates are typically observed in populations with mainly plant-based diets [5]. The high content of phytoestrogens in these plant has been proposed as the underlying factor responsible for the low breast cancer incidence in these populations but the mechanisms are poorly understood [6], [7]. One of these naturally occurring compounds is the resveratrol that has been suggested to have marked chemopreventive and chemotherapeutic properties. It is present in more than 72 plant species, including a wide variety of fruits and vegetables, such as grapes, berries, peanuts, pines and various herbs [8], [9]. The physiological function of resveratrol is to act as phytoalexin, a defensive agent protecting plants against adverse conditions such as pathogenic attack, mechanical injury, and environmental stressors [10]. In human, resveratrol exerts antitumor properties at initiation, promotion, and progression stages of carcinogenesis [11]. Resveratrol reduces cell proliferation and tumor growth, prevents chemical carcinogen-induced epithelial cell transformation [12], induces cell cycle arrest, and inhibits cell migration, invasion, metastasis and angiogenesis [13], [14]. Several studies showed that resveratrol inhibits the growth of different cancer cell lines from human oral squamous carcinoma, rhabdomyosarcoma, liver hepatocellular carcinoma, promyelocytic leukemia, as well as breast, prostate, ovarian, lung and colon cancer [15]–[25]. These effects have been associated with the ability of resveratrol to arrest cell cycle progression [26], [27], promote cell differentiation [28], and induce programmed cell death through caspase-independent or caspase-dependent apoptosis [29]–[32] and by autophagocytosis [33].

As tumors frequently develop resistance to chemotherapeutics agents, the search and clinical implementation of effective chemosensitizers still represents a pivotal goal in cancer research. Chemosensitization is one useful strategy to overcome chemoresistance. A plethora of studies reports the promising possibilities of use of dietary natural compounds to sensitize tumors to therapeutics agents [34]. Resveratrol can overcome chemoresistance in tumor cells by modulating one or various mechanisms of resistance, including apoptosis, inhibition of multidrug transporters and regulation of proteins involved in tumor cell proliferation [34]. It has been shown that resveratrol may sensitize human cancer cell lines, such as neuroblastoma, glioblastoma, breast carcinoma, prostate carcinoma, leukemia, and pancreatic carcinoma to diverse chemotherapeutic agents. However, the proteins involved in these cellular processes have not been entirely identified. Several studies have considered heat shock proteins as promising pharmacological targets for cancer therapy [35]. Notably, HSP27 is frequently overexpressed in human cancer cells resulting in apoptosis inhibition and resistance to anti-neoplastic agents including doxorubicin [36]–[38]. HSP27 overexpression confers resistance to doxorubicin in MDA-MB-231 breast cancer cells demonstrating a protective role of HSP27 against apoptosis [39].

In the present study, we searched for potential targets of resveratrol in MCF-7 breast cancer cells through a proteomic approach. We discuss the role of the resveratrol-modulated proteins in apoptosis and resistance of cancer cells to therapy. Our results about the targeted HSP27 inhibition and combinations of resveratrol with doxorubicin in the sensitization of breast cancer cells to cytotoxic therapy are also discussed.

## Results

### Resveratrol Induces Changes in Proteome of MCF-7 Breast Cancer Cells

**Figure 1:**
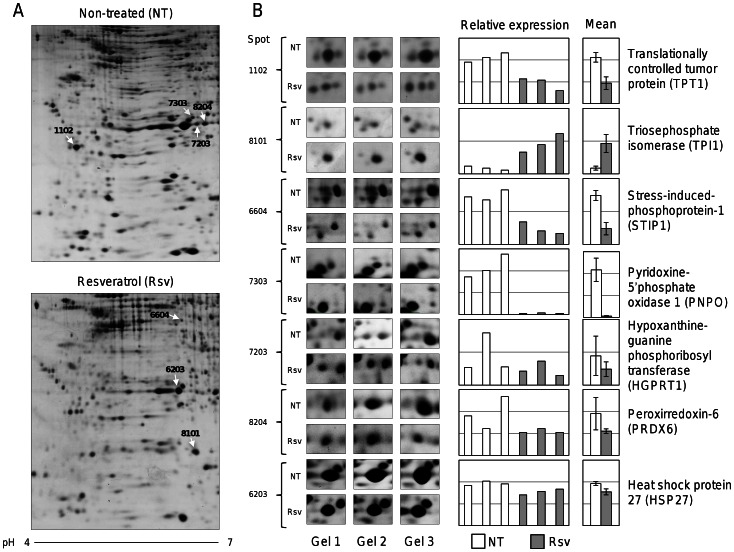
Proteomic profiles of MCF-7 breast cancer cells treated with resveratrol. (A) Representative 2DE gels of MCF-7 cells treated with 250 µM resveratrol (bottom image) or non-treated control (upper image). Protein spots selected for identification by spectrometry (ESI-MS/MS) are marked with white arrows. (B) Enlarged sections of representative 2DE gels showing differential expression of spots between resveratrol treated and non-treated cells. Right, graphs showing individual and averaged relative intensities of the selected spots. NT, non-treated cells; Rsv, resveratrol treated cells.

The specific effects of high resveratrol concentrations (250 µM) in gene expression have been previously described in MCF-7 cells [40]. Here, we evaluated the effects of resveratrol in the protein expression profiles of MCF-7 cells using a proteomic-based approach. We performed differential in gel two-dimensional electrophoresis (2DE) of total protein extracts (500 µg) from resveratrol treated and non-treated cells. MCF-7 cells monolayers growing at 70% confluence were treated with resveratrol (250 µM) or ethanol (0.3%) vehicle for 48 h. Purified proteins were loaded on 11 cm IPG strips, and separated in 2DE gels as described in Material and Methods. Gel images were captured and total spot ratios obtained from biological replicates were used for protein abundance comparisons. A representative map of proteins after 2DE analysis is illustrated in figure 1. We detected 16 differentially expressed proteins between resveratrol treated and non-treated cells (fold change >1.5; adjusted p value ≤0.05). These changes in spots abundance were consistently observed in three replicates (Figure 1B). Of these, we arbitrary selected for identification those spots that exhibited the most abundant expression in MCF-7 cells. Spots were excised from gels and digested tryptic peptides were identified using tandem mass spectrometry (ESI-MS/MS). Comparative proteomic analyses of replicates allowed the identification of seven proteins modulated by resveratrol. An overview of MS/MS peptide sequences (ion scores), Mascot scores, and sequences coverage is shown in Table 1. We identified six down-regulated proteins in resveratrol-treated MCF-7 cells corresponding to: i) heat shock protein 27 (HSP27), ii) translationally controlled tumor protein (TPT1), iii) peroxiredoxin-6 (PRDX6), iv) stress-induced-phosphoprotein 1 isoform 1 (STIP1); v) pyridoxine-5′-phosphate oxidase 1 (PNPO), and vi) hypoxanthine-guanine phosphoribosyl-transferase (HGPRT1); whereas one up-regulated protein was identified as triosephosphate isomerase (TPI1).

**Table 1:** Resveratrol modulated proteins in MCF-7 breast cancer cells identified by ESI-MS/MS mass spectrometry.

Protein name^a^ (Spot number)	Molecular mass/pI	Gene name^b^	Accession number^c^	Mascot Score	Matched peptides	Sequence coverage	MS/MS peptide sequence (ion scores)	Molecular function/Cellular process
Translationally-controlled tumor protein (1102)	19583/4.84	TPT1	P13693	507	7	71%	^39^TEGNIDDSLIGGNASAEGPEGEGTESTVITGVDIVMNHHLQETSFTK^85^ (242)^ 131^NYQFFIGENMNPDGMVALLDYR^152^ (103)^ 6^DLISHDEMFSDIYK^19^ (69)^ 111^VKPFMTGAAEQIK^123^ (81)^ 53^EDGVTPYMIFFK^164^ (53)	Calcium ion binding/Calcium binding and microtubule stabilization
Triosephosphate isomerase (8101)	26807/6.51	TPI1	P60174	533	9	78%	^18^KQSLGELIGTLNAAK^32^ (105)^ 19^QSLGELIGTLNAAK^32^ (92)^ 85^DCGATWVVLGHSER^98^ (87)^ 69^VTNGAFTGEISPGMIK^84^ (86)^ 206^IIYGGSVTGATCK^218^ (82)	Triose-phosphate isomerase activity/Gluconeogenesis, Glycolysis, Pentose shunt
Stress-induced-phosphoprotein 1 (6604)	68721/7.81	STIP1	G3XAD8	445	6	26%	^399^LAYINPDLALEEK^411^ (69)^ 125^KAAALEFLNR^134^ (62)^ 126^AAALEFLNR^134^ (57)^ 581^LMDVGLIAIR^590^ (52^ 561^DPQALSEHLK^570^ (47)^ 454^LLEFQLALK^462^ (43)	Stress-induced-phosphoprotein/Cellular network of molecular chaperones and folding catalysts.
Pyridoxine-5′-phosphate oxidase 1 (7303)	30311/6.62	PNPO	Q9NVS9	297	3	26%	^46^EAFEETHLTSLDPVK^60^ (130) ^ 164^SSQIGAVVSHQSSVIPDR^181^ (101)^ 235^GLPTGDSPLGPMTHR^249^ (74)^ 237^PTGDSPLGPMTHR^249^ (54)^ 46^EAFEETHLTSLDPVK^60^ (24)	Catalyzes oxidation/Pyridoxine biosynthesis
Hypoxanthine-guanine phosphoribosyl-transferase (7203)	24590/6.24	HGPRT1	P00492	138	4	21%	^115^VIGGDDLSTLTGK^127^ (96)^ 128^NVLIVEDIIDTGK^140^ (56)^ 141^TMQTLLSLVR^150^ (42)^34^VFIPHGLIMDR^44^ (16)	Guanine phosphoribosyltransferase activity; hypoxanthine phosphoribosyltransferase activity; magnesium ion binding; nucleotide binding/Generation of purine nucleotides
Peroxiredoxin-6 (8204)	25133/6	PRDX6	P30041	128	9	37%	^109^ELAILLGMLDPAEK^122^ (63)^ 145^LSILYPATTGR^155^ (58)^ 98^LPFPIIDDR106 (49)^ 109^ELAILLGMLDPAEKDEK125 (44)^ 85^DINAYNCEEPTEK^97^ (42)^ 133^VVFVFGPDK^141^ (41) ^156^NFDEILR^162^ (38)^ 133^VVFVFGPDKK^142^ (36)^ 183^DGDSVMVLPTIPEEEAKK^200^ (4)	Antioxidant; glutathione peroxidase; peroxiredoxin; phospholipase A2/Redox regulation; phospholipid turnover.
Heat shock protein beta-1 (6203)	22427/7.83	HSP27	P04792	257	10	60%	^172^LATQSNEITIPVTFESR^188^ (101)^ 97^VSLDVNHFAPDELTVK^112^ (74)^ 141^KYTLPPGVDPTQVSSSLSPEGTLTVEAPMPK^171^ (70) ^80^QLSSGVSEIR^89^ (64) ^28^LFDQAFGLPR^37^ (50)	Protein kinase C inhibitor activity/Stress resistance and actin organization

a,b,c Protein Knowledgebase (UniProtKB).

### Overview of Proteins Modulated by Resveratrol in MCF-7 Breast Cancer Cells

Our proteomic analysis evidenced that resveratrol directly or indirectly modulates the expression of proteins with key roles in cancer (Table 1). HSP27 is a stress-induced molecular chaperone often associated with apoptosis, drug resistance, metastasis and poor prognosis in human cancers [36]–[39]. TPT1 protein has been involved in many biological processes including cell growth, tumor reversion, and induction of pluripotent stem cells [41], [42]. PRDX6 is an antioxidant enzyme capable of reducing lipid peroxides, and it additionally possesses phospholipase A2 activity [43]. Overexpression of PRDX6 leads to a more invasive phenotype and metastatic potential in human breast cancer [44]. STIP1 is a cochaperone that organizes other chaperones [45], and it has been recently described to be secreted by human ovarian cancer cells; whereas PNPO catalyzes the oxidation of pyridoxine 5′-phosphate to pyridoxal 5′-phosphate, the active cofactor form of vitamin B(6) required by more than 140 different catalytic activities [46]. Notably, it has been described that higher plasma levels of vitamin B6 may reduce the risk for developing breast cancer [47]. The HGPRT1 enzyme participates in the purine metabolism. The lack of HGPRT1 activity causes the Lesch-Nyhan disease [48], whereas high levels of HGPRT1 have been associated to elevated synthesis of nucleotides which confers selective growth advantages to human cancer cells [49]. Finally, TPI1 is a glycolytic enzyme ubiquitously distributed in all tissues [50]. Previous studies have reported TPI1 overexpression in lung adenocarcinoma [51] and squamous cell carcinoma of the bladder [52]. Remarkably, overexpression of HSP27, TPT1, and PRXD6 proteins has been also associated to apoptosis inhibition and resistance of cancer cells to chemotherapeutic drugs [35], [41], [44], [53]–[55]. From these resveratrol-modulated proteins, we selected HSP27 for further analysis due to its implication in apoptosis inhibition and drug resistance, as well as its potential as therapeutic target [36], [56], [57].

### Resveratrol Induces Apoptosis in MCF-7 Cells

**Figure 2:**
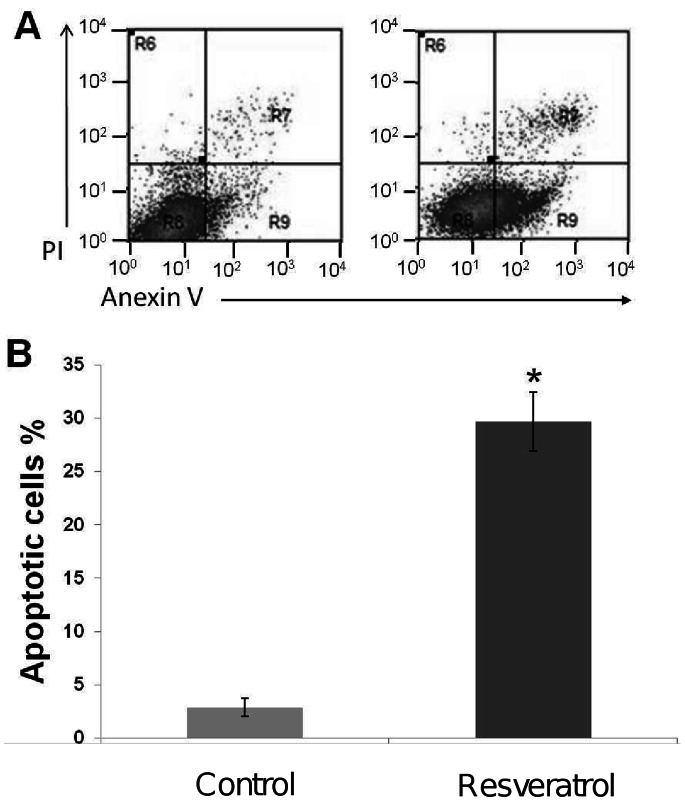
Resveratrol induces apoptosis in MCF-7 breast cancer cell lines after 48 h exposure. (A) MCF-7 cells were incubated with 250 µM resveratrol for 48 h (right panel) or non-treated (left panel). Apoptosis was evaluated by annexin V/propidium iodide dot plots. (B) Percentage of apoptotic cells in the presence of resveratrol or control (0.3% ethanol). Results shown are the mean of three independent experiments ±SD. Asterisks indicate p<0.005 compared to control.

Recently it was reported that resveratrol at 250 µM concentration has anti-proliferative effects in MCF-7 cells [40], however it was not established if this effect was a consequence of apoptosis activation. On the other hand, our proteomic findings evidenced an inhibitory effect of resveratrol on proteins whose overexpression has been related to apoptosis inhibition. Thus we sought to establish if resveratrol treatment induces apoptosis in MCF-7 cells. We quantified the number of apoptotic cells in resveratrol-treated cultures using the annexin V-FITC method. Our results showed that percentage of apoptotic cells significantly increased from 2.88% in non-treated control cells to 29.68% after 48 h treatment with resveratrol (Figure 2). These data confirm that resveratrol activates apoptosis in MCF-7 cells in agreement with previous reports in diverse human cancers using diverse resveratrol concentrations [17], [22], [24].

### Resveratrol Disrupts the Mitochondrial Membrane Potential (ΔΨm) Inducing Cytochrome c Release and Cell Death by a Caspases -9 and -3 Independent Mechanism

**Figure 3:**
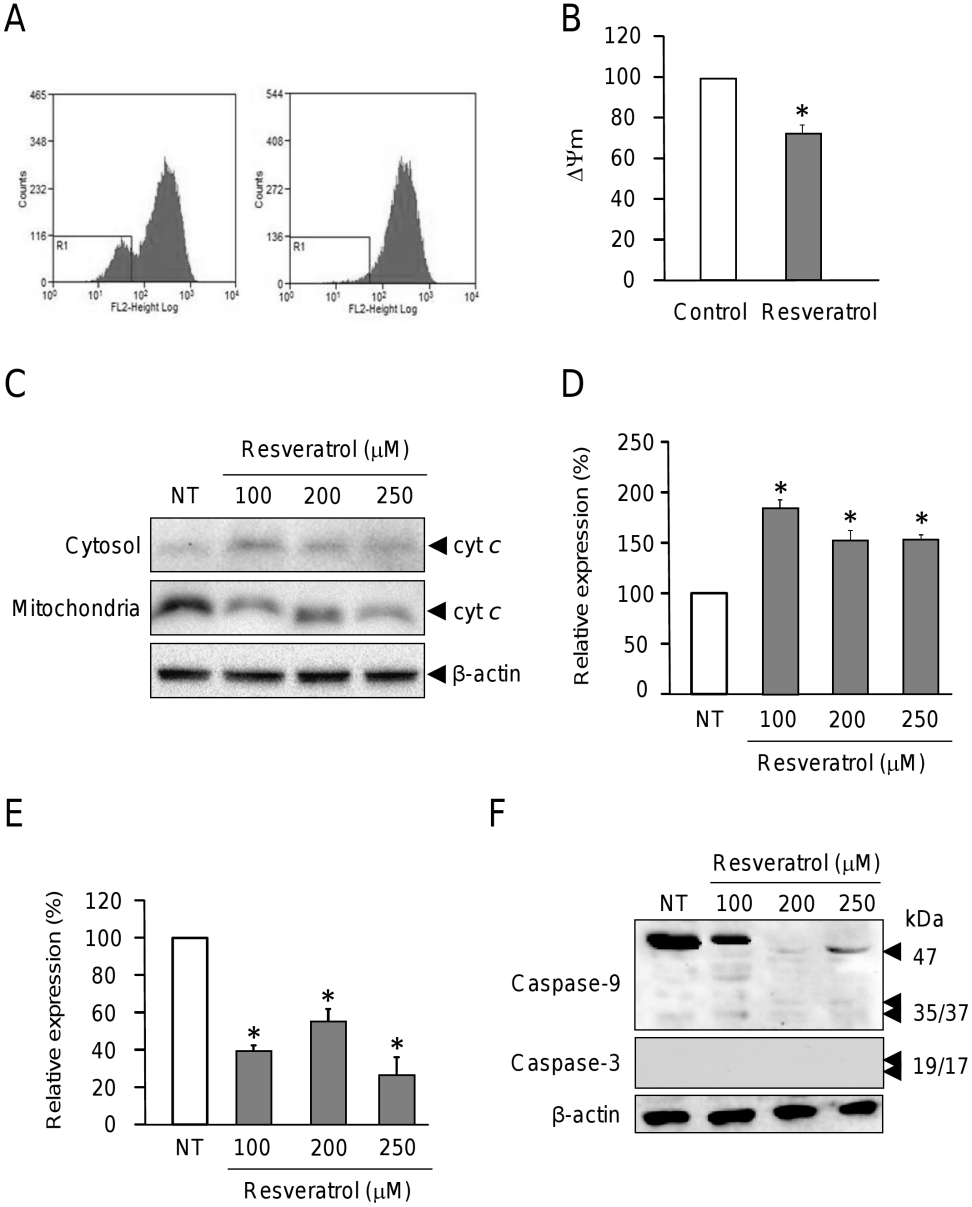
Resveratrol decreases the mitochondrial membrane potential (ΔΨm) and induces cytochrome *c* release from mitochondria. (A) Representative histograms for mitochondrial depolarization assays in MCF-7 cells after resveratrol treatment (250 µM) during 48 h (right) and non-treated (control) cells (left). Cells were incubated with TMRE (200 ng/ml) and then analyzed by FACS. (B) Bars representation of data in panel A. (C) Western blot assays of cytosolic and mitochondrial protein fractions probed with cytochrome *c* (cyt *c*) and β-actin antibodies in MCF-7 cells treated with resveratrol (100, 200 and 250 µM) or ethanol vehicle (NT) for 48 h. (D, E) Densitometric analysis of immunodetected bands in panel C for cytochrome *c* in cytoplasmic (D) and mitochondrial (E) fractions. Pixels corresponding to cytochrome *c* expression in non-treated (NT) cells were taken as 100% and used to normalize data. (F) Western blots of protein extracts from MCF-7 cells non-treated (NT) or treated with resveratrol (100, 200 and 250 µM) for 48 h using caspases -9, -3 and β-actin antibodies. Representative results are shown for Western blot assays, and densitometric data represents the mean of three independent assays ±SD. Asterisks indicate p<0.005 compared to non-treated controls.

One of the mechanisms underlying apoptosis includes perturbations in mitochondrial function. This involves a membrane depolarization represented by a decrease in membrane potential (ΔΨm) followed by an increase in permeability [58] resulting in cytochrome *c* release, apoptosome formation and caspases activation. We evaluated in more detail the involvement of mitochondria alterations in breast cancer cells treated with resveratrol for 48 h. The degree of mitochondrial depolarization was analyzed by flow cytometry in MCF-7 cells labeled with tetramethyl rhodamine ethyl ester. Our results showed that the mitochondrial membrane potential was significantly decreased by 24.65% (p<0.005) in cells treated with resveratrol (Figure 3A and B). These data suggest that resveratrol induces apoptosis in MCF-7 cells through dissipation of mitochondrial permeability. Then, we investigated the effect of increasing concentrations of resveratrol in cytochrome *c* release from mitochondria to cytosol. Treated and non-treated MCF-7 cells were submitted to differential subcellular fractionation and proteins from the cytosolic and mitochondrial compartments were analyzed by Western blot. Results indicate that MCF-7 cells treated with 100, 200 and 250 µM resveratrol exhibit a significant increase of cytochrome c in cytosol and reduced levels in mitochondrial fraction in comparison with non-treated cells (Figure 3C). However, we did not find significant differences in the amount of cytochrome *c* released to cytosol between cells treated with 200 and 250 µM of resveratrol (Figure 3D).

In order to evaluate if increased mitochondrial permeability and release of cytochrome *c* to cytoplasm results in caspases -9 and -3 activation, we performed Western blot assays in MCF-7 cells treated with 100, 200 and 250 µM resveratrol. We found that initiator caspase-9 was processed at very low levels after resveratrol treatment (Figure 3F) whereas caspase 3 was not immunodetected in MCF-7 breast cancer cells in agreement with previous studies. It has been reported that MCF-7 cells are caspase-3 negative due to mutation in coding gene [59], [60], which indicates that early mitochondrial apoptotic events may occur after resveratrol insult leading to the apoptosome formation without caspase-3 activation. An alternative mechanism of apoptosis cell death in MCF7–cells has been proposed by Sareen *et al*., [32] in which calpain activation may be responsible of proteolysis of substrates leading to apoptosis.

### Resveratrol Sensitizes MCF-7 Cells to Doxorubicin Therapy

**Figure 4:**
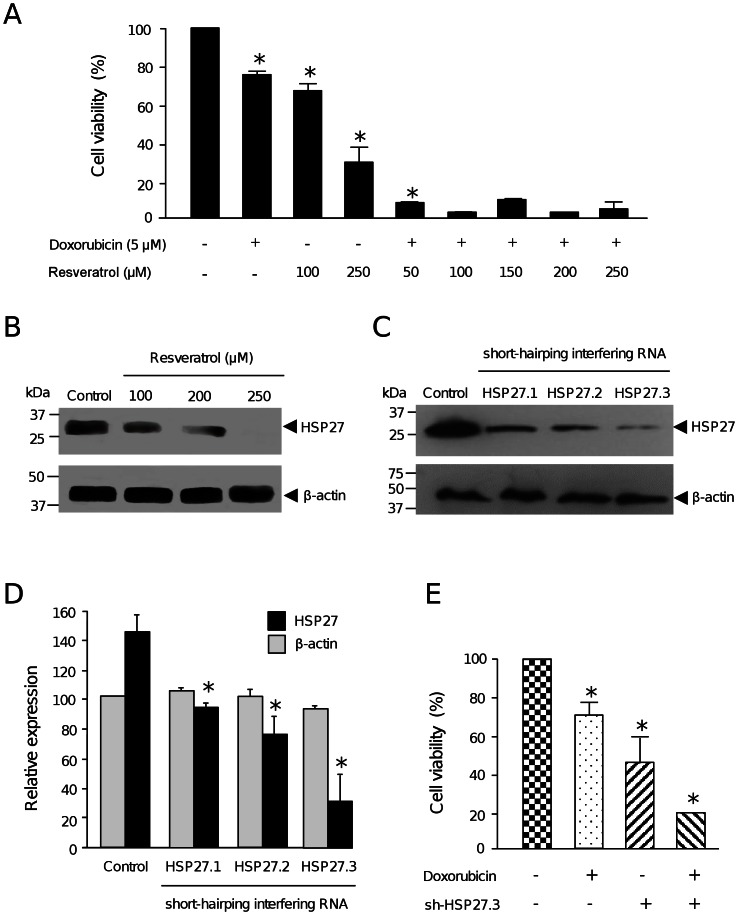
Resveratrol and HSP27 silencing sensitizes MCF-7 cells to doxorubicin treatment. (A) MTT assays of MCF-7 cells treated with either doxorobucin (5 µM) or resveratrol (100 and 250 µM) alone, or with combinations of increased concentrations of resveratrol (50, 100, 150. 200 and 250 µM) and doxorubicin (5 µM). (B) Western blot analysis of HSP27 and β-actin expression after resveratrol (100, 150, 200 and 250 µM) treatment of MCF-7 cells. (C) Western blot analysis of HSP27 and β-actin proteins in MCF-7 cells transfected with three short-harping interfering RNA constructions (pSilencer-HSP27.1, -HSP27.2 and -HSP27.3). (D) Densitometric analysis of bands shown in panel C. Pixels corresponding to β-actin expression in control (non-transfected) cells were taken as 100% and used to normalize HSP27 data. (E) Cell viability percentage of HSP27-deficient MCF-7 cells treated with doxorubicin (5 µM) for 48 h. Cell viability was determined by MTT assay. Assays were performed by triplicate and data was expressed as means ±SD. Asterisks indicate p<0.05 compared to controls.

To evaluate the potential chemosensitizing effect of resveratrol, we analyzed increasing concentrations of the polyphenol in combination with doxorubicin cytotoxic therapy. While treatments with resveratrol (100 and 250 µM) alone significantly alters breast cancer cells viability (31 and 70%, respectively), a combination of resveratrol (50, 100, 150 and 250 µM) with doxorubicin (5 µM) resulted in a markedly increase in cytotoxicity (Figure 4A). These data indicates that resveratrol efficiently sensitizes MCF-7 cells to doxorubicin therapy, at least in part by cell death induction.

### HSP27 Knockdown Sensitize MCF-7 Cells to Doxorubicin Therapy

In our proteomic analysis we observed that HSP27 was down-regulated by resveratrol treatment. Several studies have considered heat shock proteins as promising pharmacological targets for cancer therapy [61]. Notably, HSP27 is frequently overexpressed in human cancer cells resulting in apoptosis inhibition and resistance to anti-neoplastic agents including doxorubicin [62]. To evaluate the potential beneficial effects of resveratrol-mediated HSP27 inhibition in cytotoxic therapy, we first confirmed that resveratrol (100, 200, 250 µM) effectively down-regulates HSP27 using specific antibodies in Western blot assays. Results evidenced that HSP27 expression was progressively decreased in a dose manner until being undetectable at 250 µM resveratrol concentration in comparison with non-treated control cells (Figure 4B). Next, we investigated if targeted inhibition of HSP27 sensitizes the MCF-7 cells to cytotoxic therapy. HSP27 expression was abrogated by RNA interference using three specific short-harping interfering RNAs. The shHsp27.1, shHsp27.2 and shHsp72.3 sequences (Table 2) were cloned in pSilencer vector and transfected into MCF-7 cells. Results of Western blot using specific antibodies evidenced a marked down-regulation of HSP27 protein levels in MCF-7 cells transfected with the three interfering RNAs (Figure 4C). Densitometric analyses of immunodetected bands showed that shHsp27.3 was the most effective interfering RNA since it suppressed HSP27 expression by about 80% (Figure 4D); thus we selected this sequence for further RNA-based gene silencing experiments. No significant changes were observed in β-actin expression used as control.

**Table 2:** Nucleotide sequences of short-harping interfering RNAs specific for HSP27 gene.

Oligonucleotide	Sequence	Nucleotide position in HSP27 gene
shHSP27.1s	5′-GATCCATGAGACTGCCGCCAAGTATTCAAGAGATACTTGGCGGCAGTCTCATCGTTTTTTGGAAA-3	619–637 nt
shHSP27.1	5′-AGCTTTTCCAAAAAACGATGAGACTGCCGCCAAGTATCTCTTGAATACTTGGCGGCAGTCTCATG-3′	619–637 nt
shHSP27.2s	5′-GATCCGTAAAGCCTTAGCCCGGATTTCAAGAGAATCCGGGCTAAGGCTTTACTTTTTTGGAAA-3′	635–653 nt
shHSP27.2as	5′-AGCTTTTCCAAAAAAGTAAAGCCTTAGCCCGGATTCTCTTGAAATCCGGGCTAAGGCTTTACG-3′	635–653 nt
shHSP27.3s	5′-GATCCGCTGCAAAATCCGATGAGATTCAAGAGATCTCATCGGATTTTGCAGCTTTTTTGGAAA-3′	603–624 nt
shHSP27.3as	5′-AGCTTTTCCAAAAAAGCTGCAAAATCCGATGAGATCTCTTGAATCTCATCGGATTTTGCAGCG-3′	603–624 nt

Next, we evaluated if targeted HSP27 inhibition sensitizes MCF`7 cells to cytotoxic therapy. First, we showed that MCF-7 cells treated with doxorubicin for 48 h exhibited a reduction in cell viability of about 70% in comparison with non-treated cells (Figure 4E). Then, we knock-downed HSP27 by transfection of pSilencer-shHsp27.3 construct in MCF-7 cells, and determined cell viability. We observed a significant decrease (55%) of MCF-7 cells viability in comparison with non-transfected cells, indicating that HSP27 abrogation strongly alters viability of cancer cells in agreement with previous reports [61]. To evaluate if HSP27 silencing exerts synergistic effects in cytotoxicity caused by doxorubicin, shHsp27.3-transfected MCF-7 cells were incubated with drug for 48 h. Results showed a significantly decrease of about 80% in the viability of MCF-7 cells evidencing an additive effect of HSP27 silencing and drug on cytotoxicity (Figure 4E). All together these data indicate that resveratrol inhibits HSP27 resulting in a cooperative effect on doxorubicin-induced cell death.

## Discussion

Resistance of tumors to chemotherapeutics agents still represents an important challenge in cancer therapy [1]. Recent studies report the promising possibilities of use of dietary compounds to sensitize tumors to chemotherapeutics agents [3], [34]. The molecular mechanisms involved in resveratrol overcome of chemoresistance in tumor cells include the modulation of pathways involved in tumor cell proliferation, cell cycle, growth and apoptosis [34]. However, the proteins involved in these cellular processes have not been entirely identified. Our proteomic analysis evidenced that resveratrol directly or indirectly modulates proteins with key roles in cancer (Figure 1). Among these resveratrol-modulated proteins, we selected HSP27 due to its implications in several cellular events described in cancer cells including apoptosis inhibition, and drug resistance [61]–[63]. Therefore, we first demonstrated that high concentrations of resveratrol (250 µM) induce apoptosis in MCF-7 cells (Figure 2). Our data showed an increase in mitochondrial membrane permeability and cytochrome release to cytoplasm, which suggest that resveratrol-mediated apoptosis could be related to intrinsic pathway. However, we did not observe caspases -9 and -3 activation suggesting that early mitochondrial apoptotic events may occur after resveratrol insult at 200 and 250 µM leading to the apoptosome formation without the final process of caspase-3 activation. These data are agreement with several reports indicating that MCF-7 cells are caspase 3 deficient because of a point mutation in the gene [59], [60]. An alternative mechanism of apoptosis cell death in MCF-7 cells has been proposed by Sareen *et al*., [32], where calpain activation may be responsible of proteolysis of its substrates leading to apoptosis.

Previous reports indicated that doxorubicin effectiveness on breast tumor cells may be related to HSP27 expression [39], [63]. It has been reported that HSP27 is induced by hormone therapy or chemotherapy and it is able to inhibit apoptosis through multiple mechanisms [62], [63]. HSP proteins overexpression is essential for the survival of some cancers and high levels of HSP27 represent a negative prognostic marker in a wide range of tumors [35]–[39]. In addition, HSP27 overexpression confers resistance to the chemotherapeutic agent doxorubicin in MDA-MB-231 breast cancer cells, demonstrating a protective role of HSP27 against apoptosis. Moreover up-regulation of HSP27 in breast cancer cells reduces trastuzumab susceptibility by increasing HER2 stability [64]. Here we report for the first time that resveratrol mediates HSP27 down-regulation in MCF-7 cells which was associated to sensitization of cells to doxorubicin treatment. Our findings suggested that resveratrol may improve the therapeutic effects of doxorubicin probably by means of cell death induction. Many inhibitors for other members of HSP proteins family dysregulated in cancer cells, such as HSP90 have been developed and currently undergone clinical trials [65]. However, the clinical evaluation of HSP27 and HSP70 has not initiated yet. Thus, we propose that therapeutic modulation of HSP27 levels using natural compounds such as resveratrol should be further extended to animal models in order to define its potential as a clinically useful strategy for breast cancer treatment. In summary, our data indicates that resveratrol down-regulates proteins involved in apoptosis and drug resistance, providing novel targets for conducting further investigations to enhance treatment efficacy, reduce toxic side effects and overcome chemoresistance in breast cancer.

## Materials and Methods

### Cell Cultures and Treatments

Breast cancer MCF-7 cell line was cultured in Dulbecco Modified Eagle Medium (DMEM, Invitrogen) supplemented with 10% fetal bovine serum, 100 units/ml penicillin and 100 mg/ml streptomycin. Cultures were maintained in a 5% CO_2_ humidified atmosphere at 37°C. Resveratrol purchased from Sigma Chemical Co. was dissolved in ethanol to a 5 nM concentration and stored at -20°C. Treatments were carried out with resveratrol (50 to 250 µM) for 48 h. Cultures media containing fresh resveratrol (in 0.3% ethanol) were daily changed. Doxorubicin treatments were performed for 48 h in MCF-7 cells transfected with pSilencer-hsp27.3, and non-transfected (control) cells.

### Proteomics Procedures

#### Isoelectric focusing (IFE)

Proteins (500 µg) from MCF-7 cells cultured with or without resveratrol were diluted in 185 µl rehydratation solution (8 M urea, 2% CHAPS, 100 mM DTT, 0.5% v/v IPG buffer pH 3–10, and 0.002% bromophenol blue), and used to rehydrate 11 cm pH 3–10 nonlinear IPG strips (BioRad) overnight at room temperature covered with mineral oil. The strips were loaded in a Protean IEF isoelectric focusing cell (BioRad) and covered with mineral oil. Hydrated electrode wicks were mounted over the electrodes. The strips were conditioned at 250 V for 30 min at 20°C, then ramped to 8000 V over 2.5 h, maintained at 8000 V for an additional 3.5 h, and finally held at 500 V.

#### Equilibration and SDS-PAGE

After IFE, IPG strips were equilibrated for reduction and alkylation in equilibration buffer (6 M urea, 2% SDS, 0.375 M Tris-HCl pH 8.8, 20% glycerol) with 2% DTT and 2.5% iodoacetamide in first and second washes, respectively, for 10 min at room temperature. For second-dimension, proteins were resolved by 12% SDS-PAGE. Gels were run in running buffer (25 mM Tris-HCl, 192 mM glycine, 0.1% SDS) at 50 V for 20 min and 200 V until samples reach the bottom of gel. Gels were shaken gently in 75 ml fixative gel (10% methanol, 7% acetic acid) for 45 min at room temperature, rinsed with ultrapure water and incubated in 75 ml Sypro Ruby gel stain (Invitrogen). After overnight shaking stain solution was replaced with fixative gel solution. Images from 2DE gels were documented in a FLA-5100 Fuji Film scanner and adjusted using the Multigauge software. PD-Quest Advanced software version 8.0 (Bio-Rad) was used for comparative analyses. For 2DE spots selection, images of gels from resveratrol treated and non-treated cells were used to create a match-set by overlapping the triplicate gels corresponding to each sample. Automated spot detection and matching was initially used with additional manual refinement. Analysis sets were created using spots that varied quantitatively by 2.0-fold between treated and control cells. Additional sets were formed using spots whose expression varied according to a Student’s t-test with a significance level of 90%.

#### Spot cutting and proteins digestion

Spots were manually excised placed in 100 mM ammonium bicarbonate (500 µl). The solution was replaced with 500 µl acetonitrile, and vortexed for 10 min, and the gel pieces were evaporated to dryness in a SpeedVac apparatus. The gel pieces were rehydrated for 45 min in 50 µl ice cold trypsin solution (20 µg) in 760 µl 100 mM ammonium bicarbonate. The trypsin solution was removed, replaced with 50 µl ammonium bicarbonate, and the mixture incubated overnight at 37°C. 50 µl 0.1% trifluoroacetic acid in ammonium bicarbonate was added to the gel pieces and the mixture was vortexed for 15 min. The digestion liquid and rinse solutions were pooled and dehydrated. Then samples were rehydrated in 5 µl 0.1% TFA in water. Zip tips (Millipore) were used to desalt the samples following the manufacturer’s instructions.

### Tandem Mass Spectrometry (ESI-MS/MS)

Mass spectrometric analysis was carried out on a 3200 Q TRAP hybrid tandem mass spectrometer (Applied Biosystems/MDS Sciex), equipped with a nano electrospray ion source (NanoSpray II) and a MicroIonSpray II head. The instrument was coupled on-line to a nanoAcquity Ultra Performance LC system (Waters Corporations, Milford, MA). Mass calibration of the hybrid triple quadrupole linear ion trap spectrometer was done with polypropylene glycol standard solutions. The instrument was then tuned and tested using [Glu1]-fibrinopeptide B (Sigma). Samples were desalted by injection onto a Symmetry C18 UPLC trapping column (5 µm, 180 µm x 20 mm, Waters Corporations) and washed with 0.1% formic acid in 100% Milli Q water at a flow rate of 15 µl/min. Peptides were separated on a BEH, C18 UPLC column (1.7 µm, 75 µm x 100 mm, Waters Corporations) equilibrated with 2% acetonitrile, 0.1% formic acid using a linear gradient of 2–70% acetonitrile, 0.1% formic acid over a 60-min period, at a flow rate of 0.25 µl/min. Spectra were acquired in automated mode using information dependent acquisition, which involves switching from MS to MS/MS mode on detection of +2 to +4 charged species. The precursor ions were fragmented by collisionally-activated dissociation in the Q2 collision cell. Data interpretation and protein identification were performed with the MS/MS spectra data sets using the MASCOT search algorithm. Trypsin was used as the specific protease and one missed cleavage was allowed with tolerances of 0.5 Da for the precursor and 0.3 Da for the fragment ion masses. Carbamidomethyl-cysteine and methionine oxidation were used as the fixed and variable modifications, respectively. A protein ‘hit’ was accepted as a valid identification when at least two MS/MS spectrum matched at the 95% level of confidence (p<0.05). Ion score is -10*Log(P), where P is the probability that the observed match is a random event. The threshold ion score in the above conditions was 41 for p<0.05.

### Flow Cytometry Analysis

Cells were seeded at a density of 200,000 cells per well, and treated with 250 µM resveratrol for 48 h. Following treatment, cells were harvested, washed twice with pre-chilled PBS, and resuspended in 100 µl binding buffer (10 mM HEPES, 140 mM NaCl, 2.5 mM CaCl_2_). Cell suspension was mixed with 5 µl annexin V–FITC and 5 µl propidium iodide (50 µg/ml) for 10 min, washed with 500 µl binding buffer and resuspended in 300 µl PBS. Apoptosis was analyzed on the FACS Calibur flow cytometer (BDIS, Becton Dickinson). Annexin V and PI emissions were detected in the FL-1 and FL-2 channels, respectively. For each sample, data from 20,000 cells were acquired in list mode on logarithmic scales. Analysis was performed with the Summit V4.3 software. Assays were performed by triplicate.

### Mitochondrial Membrane Potential Detection Assay

Changes in the mitochondrial membrane potential were examined using flow cytometry analysis of cells stained with tetramethylrhodamine ethyl ester (TMRE, Sigma–Aldrich). After treatment with resveratrol (250 µM) for 48 h, cells were incubated with TMRE in a final concentration of 200 ng/ml for 15 min at 37°C. After washing with PBS, fluorescent intensity was monitored at 582 nm (Fluorescence 2, FL2). Assays were performed by triplicate.

### Cell Fractionation

Mitochondrial and cytosolic fractions from MCF-7 cells treated with 100, 200 and 250 µM of resveratrol or the vehicle for 48 h were obtained used the mitochondria isolation kit (Thermo Scientific USA). Briefly, pellet cells were lysed in buffer containing protease inhibitor mix (Roche, Basel, Switzerland). Homogenates were centrifuged at 700×g for 10 min at 4°C, and then supernatants were collected and centrifuged at 12,000×g for 15 min at 4°C. Resulting supernatants were cytosol fraction, while pellets were mitochondrial-enriched fractions and were washed twice in order to minimize putative contamination with cytosolic proteins. Mitochondrial proteins were obtained by add 100 µl of 2% CHAPS in TBS and vortex by 1 min. Homogenates were centrifuged at high speed for 2 min and supernatants containing soluble mitochondrial proteins were recovered and used in Western blot assays.

### Western Blots Assays

Cells were treated with resveratrol (100, 150, 200 and 250 µM) or ethanol (0.3%) vehicle for 48 h, washed with PBS, and then digested directly on the culture plates with RIPA lysis buffer (50 mM Tris–HCl, 150 mM NaCl, 1% Nonidet-40, 0.5% sodium deoxycholate, 1 mM EDTA, 1 mM PMSF) for 30 min on ice. Thirty micrograms of total protein were separated through 10% SDS-PAGE and transferred to nitrocellulose membrane. After 1h blocking with 5% non-fat milk, membrane was incubated with HSP27 (1∶1000, Abcam), caspase 9 (1∶1000, cell signaling), caspase 3 (1∶1000 cell signaling), and cytochrome *c* (1 µg/ml BD PharMingen Biosciences) antibodies overnight at 4°C. After striping, β-actin was detected in the same membrane using anti- β-actin monoclonal antibodies (1∶300, Santa Cruz Biotechnology). Secondary antibodies conjugated to horseradish peroxidase (Sigma) were used at a dilution of 1∶5000, and immunoreactivity was visualized using ECL Western blotting detection system (Pierce). Densitometric analysis of immunodetected bands was performed using the Syngen Image Software.

### Design of Short-harping Interfering RNAs

Three specific sequences (Table 2) targeting the HSP27 gene were designed and cloned in pSilencer 2.1-U6 vector (Ambion). pSilencer-HSP27 constructions contain a U6 promoter followed by a 19–22-nt sense strand of HSP27 small interfering RNA sequences, a 9-nt loop (5′-TTCAAGAGA-3′), a 19–22-nt antisense strand of siRNA, and a stretch of six deoxythymidines. After PCR amplification of inserts and digestion with *Bam*HI and *Hind*III, the three fragments were inserted into pSilencer-2.1-U6 vector resulting in pSilencer-shHSP27.1, -shHSP27.2, and -shHSP27.3 plasmids. Constructions were automatically sequenced to confirm sequences identity.

### Cell Transfections

Transfections were done using lipofectamine 2000 (Invitrogen). 1×10^5^ cells were cultured in 24-well dish plates for 24 h at 37°C. Cells were mixed with plasmid constructions (1 µg) in Opti-MEM medium (Invitrogen) and incubated for 5 min at room temperature. Lipofectamine 2000 mixed with Opti-MEM was gently homogenized and incubated again for 30 min at room temperature. Samples were added to each well, mixed gently by rocking the plate for 2 min and fresh Opti-MEM medium (250 µl) was added. Transfected cells were incubated at 37°C for 4 h. Finally the medium was removed and DMEM-10% Glutamax medium (Invitrogen) was added. Cells were incubated at 37°C for 48 h.

### MTT Assays

MTT tetrazolium salt colorimetric assay was performed as follows. MCF-7 cells were plated at a density of 2×10^5^ cells in cell culture dishes. Then, cells were incubated for 48 h with doxorubicin (5 µM) in the presence or absence of resveratrol (100, 150, 200 and 250 µM) as previously reported [35]. At the end of the treatment period, cells were incubated in 3-[4,5-dimethylthiazol-2-yl]-2, 5-diphenyl tetrazolium bromide (MTT, 0.5 mg/ml) for 30 min. The medium was removed and formazan dye crystals were solubilized with 500 µL isopropanol. Absorbance was measured in a spectrophotometer apparatus at 540 nm wavelength (Bio-Rad). The percentage of growth was calculated using as 100% the value corresponding to control cells treated with 0.3% ethanol. Assays were performed by triplicate.

### Statistical Analysis

Each experiment was performed at least three times, and the results were presented as mean ± S.D. One-way analysis of variance (ANOVA) followed by Tukey’s test were used to compare the differences between means. A p<0.05 was considered as statistically significant.
